# Using Twitter Comments to Understand People’s Experiences of UK Health Care During the COVID-19 Pandemic: Thematic and Sentiment Analysis

**DOI:** 10.2196/31101

**Published:** 2021-10-25

**Authors:** Esther Ainley, Cara Witwicki, Amy Tallett, Chris Graham

**Affiliations:** 1 Picker Institute Europe Oxford United Kingdom

**Keywords:** patient experience, COVID-19, remote health care, phone consultation, video consultation, Twitter, sentiment analysis, social media, digital health, public health, public opinion

## Abstract

**Background:**

The COVID-19 pandemic has led to changes in health service utilization patterns and a rapid rise in care being delivered remotely. However, there has been little published research examining patients’ experiences of accessing remote consultations since COVID-19. Such research is important as remote methods for delivering some care may be maintained in the future.

**Objective:**

The aim of this study was to use content from Twitter to understand discourse around health and care delivery in the United Kingdom as a result of COVID-19, focusing on Twitter users’ views on and attitudes toward care being delivered remotely.

**Methods:**

Tweets posted from the United Kingdom between January 2018 and October 2020 were extracted using the Twitter application programming interface. A total of 1408 tweets across three search terms were extracted into Excel; 161 tweets were removed following deduplication and 610 were identified as irrelevant to the research question. The remaining relevant tweets (N=637) were coded into categories using NVivo software, and assigned a positive, neutral, or negative sentiment. To examine views of remote care over time, the coded data were imported back into Excel so that each tweet was associated with both a theme and sentiment.

**Results:**

The volume of tweets on remote care delivery increased markedly following the COVID-19 outbreak. Five main themes were identified in the tweets: access to remote care (n=267), quality of remote care (n=130), anticipation of remote care (n=39), online booking and asynchronous communication (n=85), and publicizing changes to services or care delivery (n=160). Mixed public attitudes and experiences to the changes in service delivery were found. The proportion of positive tweets regarding access to, and quality of, remote care was higher in the immediate period following the COVID-19 outbreak (March-May 2020) when compared to the time before COVID-19 onset and the time when restrictions from the first lockdown eased (June-October 2020).

**Conclusions:**

Using Twitter data to address our research questions proved beneficial for providing rapid access to Twitter users’ attitudes to remote care delivery at a time when it would have been difficult to conduct primary research due to COVID-19. This approach allowed us to examine the discourse on remote care over a relatively long period and to explore shifting attitudes of Twitter users at a time of rapid changes in care delivery. The mixed attitudes toward remote care highlight the importance for patients to have a choice over the type of consultation that best suits their needs, and to ensure that the increased use of technology for delivering care does not become a barrier for some. The finding that overall sentiment about remote care was more positive in the early stages of the pandemic but has since declined emphasizes the need for a continued examination of people’s preference, particularly if remote appointments are likely to remain central to health care delivery.

## Introduction

The COVID-19 pandemic has presented many challenges to health and care services. New methods of care delivery have been rapidly introduced to create capacity in hospitals, enable health care professionals to work remotely, and to reduce the risk of transmitting the virus in care settings [1]. Care was adapted at speed, and people had to rapidly learn new ways of navigating the health and social care system, such as accessing care remotely. Since the onset of COVID-19 in spring 2020, general practitioner (GP) practices have provided a much higher proportion of consultations by phone, although not by video consultation. In 2020, the proportion of phone consultations increased from 14% in February to 28% in March and then stabilized at 48% between April and June [2]. In contrast, the proportion of online/video appointments (including nonvideo-based online consultations such as live chat or internet telephony [VoIP]) remained at less than 1% over the same period [2]. However, the quality of these data is likely to be impacted by variations in the approach to appointment management between practices, and it is suggested that many video consultations start as a telephone appointment and then switch to video, which therefore may be undercounted [2]. The uptake of video consultation (Near Me) in general practice within Scotland increased markedly from 38 consultations in February 2020 to a peak of 14,602 in May 2020, although it then decreased by 20% in June-August 2020 when lockdown restrictions eased [3].

Although research prior to the COVID-19 outbreak has examined patients’ experiences of receiving care remotely, it is argued that the findings may not be applicable to the current climate where services are being impacted by COVID-19 [4]. Several studies have examined the impact of COVID-19 on service delivery changes, but there has been little published research examining patients’ experiences of accessing remote consultations since the COVID-19 pandemic. A patient survey conducted by Oxleas National Health Service (NHS) Foundation Trust showed that the “convenience” of video consultations was the main theme that arose [5]. It is vital that such research is undertaken to understand and learn directly from people’s experiences, particularly as remote methods for delivering care are likely to be maintained [1].

Social media sites such as Twitter provide opportunities for research to understand how people are experiencing care. Twitter data can be useful for exploring people’s opinions on health issues or treatment [6-9], insights into previous pandemics [10,11], and public reactions to the COVID-19 outbreak [12-14]. There are both strengths and limitations of using Twitter data as a source for research. One advantage is that it allows quick and relatively easy access to people’s views on particular topics, and the data can be used without obtaining explicit informed consent since it is part of the public domain [15,16]. Moreover, Twitter data are useful to be able to explore people’s views when it may be inappropriate and difficult to conduct primary research. Research using Twitter may also allow the voices of people who may be more critical of services to be heard, which may be missed if only face-to-face methods are used [17]. However, using Twitter as a source of data is limited to those with access to the internet and who engage in this particular social media platform. Furthermore, there is evidence that British Twitter users are not representative of the general population; they are generally younger, wealthier, and better educated [18,19]. This means that caution should be taken when extrapolating the findings from this research to the wider population.

The aim of the study was to use content from Twitter to understand discourse around health and care delivery in the United Kingdom as a result of COVID-19, focusing on the views and attitudes related to care being delivered remotely (including through video consultations and telephone calls, as well as other innovative methods).

## Methods

### Identification and Collection of Tweets

Three search terms were used to collect relevant tweets to address the research objective (Table 1). For each search term, the following criteria were specified: date range, January 1, 2018 to October 10, 2020 (date of extraction); location, restrict to the United Kingdom; and language, English.

**Table 1:** Number of tweets extracted from Twitter for each search term.

Search term	Number of tweets extracted
[Video|Virtual|Remote|*phone|Telehealth|Telecare|Online AND Consultation|Appointment AND GP|Doctor|Dr]	764
[“Video|Virtual|Remote|*phone|Telehealth|Telecare|Online Consultation|Appointment” AND Care|NHS|Nurse|Physiotherapist|“Occupational therapist”|Chiropodist|Podiatrist|“Health visitor”|Dietician]	494
[Video|Virtual|Remote|*phone|Telehealth|Telecare|Online AND Consultation|Appointment AND “chronic|ongoing condition”|Hypertension|“High Blood Pressure”|Depression|Diabetes|Asthma| “Kidney disease”|Heart|Cardiovascular|Cancer|COPD|“Chronic obstructive pulmonary disease”|Stroke|“mental health”]	150
Total	1408

The third search term sought to extract tweets posted by or referring to people with long-term conditions to understand their experiences of remote care. The names of specific long-term conditions were included in the search term rather than more general terms such as “long-term condition” or “chronic condition” that are less likely to be used in tweets. These were based on the most prevalent conditions in England reported in the Quality and Outcome Framework [20].

Twitter data acquisition was achieved using a scraper written in Python 3, which interfaced with the official Twitter search application programming interface (API). Search terms and specifications were converted into Twitter API query language. The scraper made requests to the API for data fitting a particular set of criteria as outlined in the research brief, and then would scroll through that data, writing it to files for delivery and processing. To restrict the search to the United Kingdom, “place” information (a form of geographic tagging) was used to restrict to UK countries. This was a more favorable approach than using longitude and latitude data, which might include tweets posted outside the United Kingdom (such as parts of France or the Republic of Ireland) or exclude areas that should be included (such as the Isle of Wight).

A total of 1408 comments across the three search terms (detailed in Table 1) were extracted. In addition to the tweet text, the following metadata were acquired: date the tweet was posted; username and ID; tweet ID; numbers of Likes, retweets, replies; user bio information (eg, user description, user follower count, geographical location [“place ID” and “place name”]).

### Data Cleaning and Analysis

Tweets and the associated metadata extracted from the scrape were imported into Excel. Of the 1408 tweets extracted, 161 duplicate tweets across the search terms were identified via the unique tweet identification number. After removal of duplicates, 1247 comments remained. The tweets and dates posted were imported into NVivo software for manual coding. To develop the coding frame, two researchers analyzed a sample of 300 comments each and coded them thematically, using an inductive and deductive approach to coding. An initial codebook was discussed and agreed upon. This was revised following further coding and additional nodes were added to the codebook when new topics were identified. After final development of the codebook, Cohen κ scores were calculated both for the primary theme coding and sublevel coding between the two researchers for 200 jointly coded tweets. This showed a very high level of agreement for the primary theme coding (κ=0.93) and a good level of agreement for the sublevel coding (κ=0.76).

Seven percent of the tweets were coded to more than one theme. Many of the codes had a positive, neutral, and negative subcategory to aid comparison across different types of remote care delivery and to understand sentiment. The tweets assigned to a neutral sentiment referred to remote care without any opinions expressed (such as people stating that they had accessed/attended a telephone appointment).

Following the coding process, both researchers examined the tweets assigned to each of the codes and grouped comments into key themes. These themes were then analyzed to identify topics and patterns in the data.

### Identifying Irrelevant Comments

During the manual coding, 610/1247 (48.92%) tweets were identified as irrelevant to the research question and were coded as “unusable.” These comments were varied in nature and covered a range of topics (see Textbox 1).

A total of 637 tweets were included in the analysis following the removal of duplicate and irrelevant tweets.

**Types of tweets identified as irrelevant to the research objective.**

- Tweets about accessing general practitioner appointments that did not refer to remote care (most of these were posted before the pandemic and appeared to refer to face-to-face consultations)
- Tweets about COVID-19 that were not directly related to the research question, such as people tweeting about their symptoms, the National Health Service test and trace service, or the virus in general
- Health-related tweets but not about people’s views or experiences of care and/or how these have been impacted as a result of COVID-19
- Nonhealth-related tweets such as tweets referring to virtual appointments for British Gas, hair salons, etc
- Tweets that included words such as “online” or “video,” but were not relevant to the research question, such as those referring to people watching health-related videos or health care providers reminding patients to book flu vaccines online
- Tweets that could not be understood out of context, such as replies to tweets that made little sense on their own

## Results

### Overall Frequency and Sentiment of Tweets

Table 2 displays the frequency of tweets by month that referred to remote care, showing that the COVID-19 outbreak increased the discourse on remote care delivery. Based on the three search terms, there was an average of 10 monthly tweets between January 2018 and February 2020, compared with a monthly average of 50 tweets between March and September 2020 (October was not included in this calculation as the Twitter data were only extracted up until October 10). There was a sharp rise in the number of tweets in March 2020 when the United Kingdom first went into a country-wide lockdown at the onset of the pandemic.

**Table 2:** Number and percentage of tweets referencing remote care over time (N=637).

Year		Tweets, n (%)
**2018**		
	January	8 (1.3)
	February	11 (1.7)
	March	7 (1.1)
	April	8 (1.3)
	May	7 (1.1)
	June	16 (2.5)
	July	6 (0.9)
	August	8 (1.3)
	September	13 (2.0)
	October	13 (2.0)
	November	9 (1.4)
	December	3 (0.5)
**2019**		
	January	18 (2.8)
	February	7 (1.1)
	March	6 (0.9)
	April	6 (0.9)
	May	8 (1.3)
	June	4 (0.6)
	July	6 (0.9)
	August	15 (2.4)
	September	13 (2.0)
	October	10 (1.6)
	November	9 (1.4)
	December	8 (1.3)
**2020**		
	January	16 (2.5)
	February	17 (2.7)
	March	90 (14.1)
	April	36 (5.7)
	May	48 (7.5)
	June	27 (4.2)
	July	42 (6.6)
	August	55 (8.6)
	September	54 (8.5)
	October	33 (5.2)

### Thematic Analysis

#### Overall Themes

There were five main themes identified in the tweets extracted, which are summarized in Textbox 2.

The largest number of tweets were related to accessing remote care. As some tweets were coded under more than one theme, the total number of tweets used in the thematic analysis (N=681) is greater than the overall number of tweets in the dataset (N=637).

**Main themes identified.**

**1. Access to remote care appointments (267/681 tweets, 39.2%)**

Views on accessing phone or video appointments, including the ease/difficulty of getting an appointment

**2. Quality of remote care delivery (130/681 tweets, 19.1%)**

Views/experiences on the standard of care provided and the nature of the interaction with health care professionals

**3. Anticipation of remote care (39/681 tweets, 5.7%)**

Views and attitudes toward remote care appointments ahead of receiving such care

**4. Online booking and asynchronous communication (85/681 tweets, 12.5%)**

Attitudes/experiences of using online appointment booking systems or asynchronous approaches to communicating with health care professionals (eg, messaging systems)

**5. Publicizing changes to services or care delivery (160/681 tweets, 23.5%)**

Tweets publicizing remote ways of delivering care or informing people of changes to care as a result of COVID-19

The “online booking/asynchronous communication” and “publicizing changes to care” themes are not discussed in this paper as they were considered to be less relevant to this research, which is focused on the views to care being delivered remotely. Each of the other themes is discussed in more detail below.

#### Access to Remote Care

##### Proportion of Tweets Related to Theme

Of the 267 tweets that were related to accessing care remotely, those referring to phone consultations accounted for 81.3% (n=217), with video/online consultations accounting for 18.4% (n=49) of tweets. One tweet (0.4%) referred to accessing both a phone and video consultation. Comments were posted about accessing phone or video appointments both before and since COVID-19, although the number of tweets increased markedly since March 2020 in a similar pattern to that observed with all coded comments (Table 2). Based on our search terms, there was an average of 3 monthly tweets on accessing remote care appointments between January 2018 and February 2020, compared with an average of 24 tweets a month between March and September 2020.

##### Sentiment

The sentiment of tweets coded in this theme were mixed, with a similar proportion of positive (84/267, 31.5%), neutral (77/267, 28.8%), and negative (106/267, 39.7%) comments. To identify any changes over time, the sentiment of tweets was compared across three time periods: before COVID-19 (January 2018-February 2020), in the early stages of the pandemic (March-May 2020), and in the following stages of the pandemic when some UK restrictions had been eased (June-October 2020).

**Figure 1:**
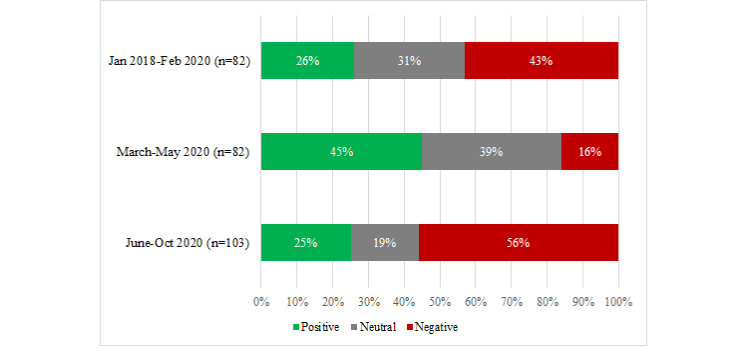
The proportion of positive, neutral and negative tweets on access to remote care at different time periods (n=267).

The overall sentiment of these tweets changed at different time periods (Figure 1). During the initial stages of the pandemic, there was almost double the proportion of comments with a positive tone (37/82, 45%) when compared with those posted in the time periods both before COVID-19 (21/82, 26%) and subsequently when some of the restrictions had been lifted (26/103, 25.2%).

Some tweets posted in the later period (June-October 2020) highlighted people’s frustrations that only remote care appointments were still being offered, despite restrictions having eased across the United Kingdom: “Can still only get a phone appointment with the dr yet people in service industries been back on the front line since July” [September 2020].

In terms of the positive tweets posted in the initial period following COVID-19 (March-May 2020), there were some tweets that expressed people’s gratitude to receiving an appointment, which were not as evident in the later time period: “Very grateful to have just had my respiratory consultant appointment by telephone this afternoon” [April 2020].

Possible explanations for the overall change in sentiment shown in Figure 1 are considered further in the Discussion. Within the overarching theme on “access to remote care,” two key subthemes were identified: ease/difficulty of getting a telephone/video appointment (including the use of remote appointments as a preliminary to a face-to-face consultation) and the lack of specific phone appointment timings.

###### Ease/Difficulty of Accessing a Remote Consultation

Tweets that were *positive* about accessing phone or video appointments centered on the efficiency of the remote care service, such as the speed of booking and “attending” an appointment, and the convenience of not needing to travel to a GP practice or wait for the appointment in the surgery.

Had to speak to my GP about a minor thing this morning and v impressed - called to arrange a phone appointment which was set up within about an hour - then could do a video call from a browser on my phone to do an examination. Hope this is something they’ll continue to offer

May 2020

Some tweets also reflected positively on the safety of accessing care remotely during the pandemic:

I've just had a very interesting video consultation with Dr [name]. (My GP) We were using AccRX. This is a brilliant use of technology which means I don't have to go down to the practice. Especially useful during the current Coronovirus situation. Brilliant!

March 2020

In contrast, tweets that were *negative* in tone within this subtheme highlighted people’s frustrations with needing to wait a long time for a phone appointment, both prior to and since COVID-19.

Once answered our surgery then tells us a Dr will phone us back within 5 days, and then if you're lucky the appointment may be a month away. Shocking!

January 2019

Phoned GP for appointment. Not doing appointments at this time. I can have a phone consult on 13th July!!! [2 weeks later].I'm in pain now or I wouldn't have called #NHS

July 2020

The difficulty in actually being able to book a phone consultation was also mentioned in several tweets due to lengthy waiting times to get through to the GP practice initially (ie, phone queues), the practice of GP receptionists triaging patients first, and phone consultations being carried out as a preliminary to a face-to-face appointment: “Patients, who telephone a GP Surgery, may have to submit to a ‘telephone interrogation’ by...a receptionist...before any appointment is arranged” [July 2020].

###### Lack of Specific Appointment Times

Some of the negative tweets about accessing remote appointments (particularly phone) were around the lack of specific appointment times or appointments running late. Such tweets were posted both prior to and since the COVID-19 pandemic.

I dunno why GP surgeries are using COVID as an excuse to be even more useless. I’ve just spent 18 minutes of my 30 minute lunch break making 44 phone calls to get an appointment. Have to ring at this exact time. In return they’ve given me a 2.5 hour slot they might call back in.

September 2020

Some people expressed their frustration that not having a specific time for a phone consultation impacted their ability to carry out daily activities, with a particular reference to confidentiality of discussions:

Waited weeks for a phone appointment with the doctor to discuss PCOS diagnosis. Of course I just got the Spanish inquisition about my ovaries in the queue for Morrisons. Soz queue.

June 2020

##### Quality of Remote Care

###### Proportion of Tweets Related to Theme

Tweets were coded in this theme if the quality of care/service received via phone or video consultations was mentioned, including the patients’ interaction with health care professionals. Just over half of the tweets coded in this theme were positive in sentiment (67/130, 51.5%). Although remote consultations were being carried out before COVID-19, only 16.9% (22/130) of the tweets extracted from our search terms discussed the quality of the care delivered via phone/video before March 2020.

###### Sentiment

**Figure 2:**
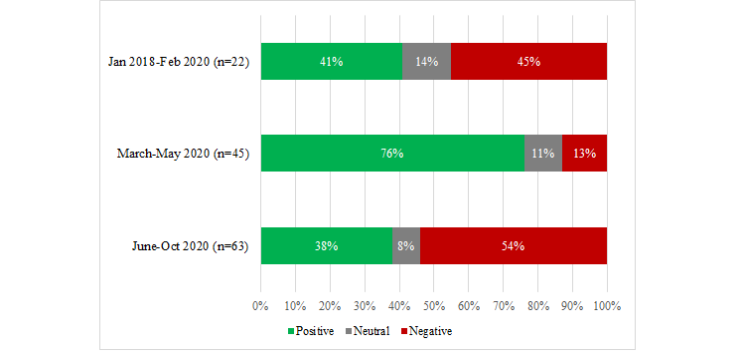
The proportion of positive, neutral and negative tweets on the quality of remote care at different time periods (n=130).

The sentiment of tweets relating to care quality changed at different time periods in a similar pattern to that noted previously for tweets about accessing remote care. During the initial stages of the pandemic (March-May 2020), there was double the proportion of comments with a positive tone when compared with the time period when some restrictions had been lifted (June-Oct 2020), and almost double the proportion of positive tweets in March-May 2020 (34/45, 76%) when compared with the prepandemic time period (Figure 2). Possible reasons for the change in sentiment noted at these two themes are considered in the Discussion.

The key subcategories identified in this theme were: efficacy of prescribing (positive comments only), standard of the care provided, and the nature of the interaction with health care professionals.

##### Efficient Prescribing

The most common positive subcategory, accounting for over one third of the positive tweets in the quality of remote care theme, was on prescribing. Tweets referred to the ease and efficiency at which prescriptions had been issued and/or received following their remote appointment. Almost all of these tweets were posted in the early stages of the COVID-19 pandemic (March-May 2020). Some tweets specifically noted that prescribing medications via remote consultations was an improvement to traditional face-to-face consultations and that this way of delivering care should be retained following the pandemic.

I had a speedy consultation. Sent photos in advance. A quick phone call & prescription sent to my local pharmacy. Saved me and the GP loads of time. This so should be the new normal.

April 2020

##### Standard of Care

There were several *positive* tweets about the quality of the care received via remote consultations. Some tweets were quite general and simply referred to a good service or a positive experience. Other tweets specified how the quality of care was good such as obtaining a quick referral to secondary care and receiving follow-up information.

Big shout out to the Trauma Physios at the [hospital] today. I had my physio appointment on the phone and an email sent with a list of exercises. I was also given another appointment in 6 weeks which could be audio visual. Fantastic service. The whole NHS are amazing.

March 2020

The advantage of remote consultations for people with long term conditions was also noted in a small number of tweets:

Had my first hospital consultation online with a Dr at [name]
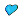
such an incredible experience during these difficult times, think it is going to be a way forward for people with #chronicillness Ok, now I need to attend hospital for tests, scans etc but it was less stressful.

May 2020

Being able to link to other patient data through technology during a remote consultation was also noted as an advantage in a few tweets:

It does depend on the type. My video appt with my diabetes consultant was great as I can download my insulin pump info plus daily blood tests so we could look at it together and do what was necessary. Fab appointment & no hanging about!

August 2020

Tweets with a *negative sentiment* in this theme were mainly in relation to phone rather than video appointments. Some people felt that not seeing a health care professional face-to-face provided a less thorough consultation; a lack of visuals and/or not being physically examined were noted as issues by some:

…All I could get was a telephone consultation which was alright to a point, but he can't see the area where the trouble is. When I asked would he make physical appointment he said they were emergency only, but I could send pictures by email
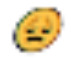

July 2020

It was implied in some tweets that the level of care or treatment received via a phone consultation was inadequate or had not fully resolved the health problem. Other tweets expressed concern and frustration that phone consultations had resulted in the condition being incorrectly treated or diagnosed by GPs:

My 94yo mum finally got an appointment with consultant after being in agony for 13 weeks. GP would only do phone consult and wouldn't refer her, just gave out morphine. Turns out her leg is broken and displaced and her hip is fractured. Her operation is on Wednesday

June 2020

Some tweets were more general in nature with people expressing their reservations about the care provided via phone appointments when compared with face-to-face interactions with health care professionals. For instance, one person considered the change from a face-to-face appointment to a phone appointment with a hospital consultant a “downgrade”:

four weeks to give me the anti clot injection. I have been troubled with high potassium levels but this was dealt with arms length by my GP. I was supposed to see my surgeon next week but this has been downgraded to a telephone consultation. I am beginning feel so alone

June 2020

A small number of tweets (both before and since COVID-19) also questioned the quality of care that could be provided via video consultation, with concerns that it could not offer the same standard of care as a face-to-face consultation. Interestingly, a mixed experience of remote care delivery was noted by one person who reflected that it may not be appropriate for all health issues:

When I scratched my arm in the garden, I had video consultation. GP sent prescription for antibacterial cream to pharmacy near me. That worked well. Practice nurse did asthma review over phone which seemed odd -not sure if that's really the best way. Smear test next week...

August 2020

##### Interaction With Health Care Professionals

Tweets from people that were *positive* about the interaction with health care professionals during remote consultations noted good interpersonal skills of the health professional (listening, helpful, and reassuring) in addition to their professionalism.

My virtual appointment today was actually the best appointment I've had in years. The dietician is going to contact me as well as the nurse to help me combat the hypos and my gastroparesis. It was great having a Dr listen to me #type1diabetes #gbdoc

May 2020

*Negative* tweets about the interaction with health care professionals referred to phone appointments being rushed and/or a lack of interest shown by the health care professional.

Seeing a doctor? Fat chance. A brief rushed phone consultation. No examination. And for the flu jab, guess what? It’s being done as a drive-through in the car park. I might just say thanks, but no thanks. But I’m nearly 76.

August 2020

There were also a small number of tweets that noted some functional issues associated with video consultations that impacted the quality of care provided:

My son had a follow up appointment via a video chat from the hospital where the doctor said she couldn’t see as the picture was out of focus.

August 2020

Although some of the tweets in this theme implied that the poor experiences were due to the care being delivered remotely, others appeared to be more related to the doctor’s knowledge and/or interpersonal skills, which could be the same when delivering care face-to-face.

#### Anticipation of Remote Care

This theme captured people’s views of remote care appointments before they had actually received them. This was not one of the main themes to emerge from the data, with only 39 comments coded under it and 79% of these tweets were only posted since March 2020. Many tweets under this theme were written in either a curious or sarcastic manner, possibly due to a lack of explanation or understanding of how a telephone/video appointment could work: “Receptionist at the GP surgery booked me in for a phone appointment. For my blood test.” [June 2020]

Reservation was expressed by some people of how a remote care appointment could work effectively when they felt a physical examination was needed for their particular condition/health problem:

tried making an appointment at the dr for [name’s] rash and it’s a telephone appointment
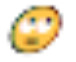
now I’m no expert but surely the dr needs to actually see the rash
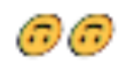

June 2020

6 month cancer check soon, letter from hospital today saying it will be a telephone appointment! How’s that going to work? Also referred to hospital by my GP for a throat problem, they’re giving me a telephone appointment for that! Went to the dentist last week and had a filling

July 2020

Some people suggested in their tweets that remote appointments were potentially a waste of time, when they knew they would need a physical examination or procedure anyway, such as vaccinations and blood tests. There was a view expressed in a small number of tweets that moving to remote care appointments would put people’s health at risk:

Sorry but how can you do a examination / consultation over the phone, appointment cancelled in August due to COVID, you are putting cancer patients at risk, and leaving them to fend for themselves #FeelingLetDown #COVID19 #AtRisk #CancerPatients2nd #melanoma

July 2020

Concern was also expressed in some tweets about anticipating bad news about their, or their family members’, health or condition over the phone rather than in a face-to-face appointment:

I had a few blood tests taken 2 days ago and now the doctor surgery have phoned to say I have to arrange a telephone consultation about the results. I'm left here thinking like “what if there's something bad wrong with me?!”
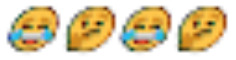

May 2018

Although most tweets in this theme were negative in tone, there were tweets where people were supportive of the need to carry out their appointment remotely due to COVID-19:

So my cancer follow up appointment on Monday to check bloods and neck is to be done over the phone. must be some new technology I’m not aware of but to be fair good decision as it’s non urgent so
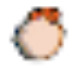
good on you [name] hospital. #NHSheroes

March 2020

My Acute asthma appointment is now a telephone appointment rather than F2F #CommonSense wins #cornoravirusuk

March 2020

There were also some tweets where people were positively anticipating their remote appointment and were appreciative that this form of care delivery was now a possibility. One person mentioned how they had been apprehensive about remote appointments, but after having had their first, was really reassured:

Super excited to be waiting for my video appointment with the [hospital name]! I remember back in the day when this was all a future reality 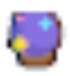

July 2020

## Discussion

### Principal Findings

This study explored tweets about remote care delivery between January 2018 and October 2020 in the United Kingdom, and showed that the volume of related tweets increased markedly following the COVID-19 outbreak. The key themes identified in the tweets were access to remote care, quality of remote care, and anticipation of remote care. Mixed public attitudes and experiences to the changes in service delivery were found. The proportion of positive tweets regarding access to, and quality of, remote care was higher in the immediate period following the COVID-19 outbreak (March-May 2020) when compared to the time before COVID-19 onset, and the time when restrictions from the first lockdown eased (June-October 2020).

This research showed that discourse about remote health care delivery increased markedly on Twitter since the onset of the COVID-19 pandemic in March 2020. This finding is perhaps unsurprising given the changes to how services have been delivered. The pandemic has resulted in the rapid adoption of digital technology and has revolutionized the use of remote care [1,21,22]. Our research allowed us to use online data to explore how Twitter users have communicated about changes in care delivery during a time when it would have been very difficult to conduct primary data collection with patients.

Although the search terms were not restricted to primary care, and included the search terms “doctor” and “dr” in addition to “GP,” the majority of the tweets extracted were about GP consultations. This is perhaps unsurprising given that primary care is the first point of contact in the health care system for people seeking advice or treatment. There was also a much higher proportion of tweets about phone consultations rather than other types of remote care delivery such as video consultations or online messaging systems. This reflects remote primary care delivery patterns since the onset of COVID-19 in March 2020, with GP practices providing a much higher proportion of phone rather than video consultations [2]. It has been suggested that the low proportion of video consultations in general practice may, in part, be due to the limited usefulness of video consultations over telephone or face-to-face in most cases [23]. Although the uptake of primary care video consultation in Scotland increased significantly in response to COVID-19, an evaluation of the program noted variation in uptake by GP practices both within and between NHS boards, and there was also a fall in usage between June and August 2020 [3]. The reasons suggested for the limited use, and fall in usage, of video consultations across GP services in Scotland include the case mix (where telephone was sufficient for dealing with straightforward problems in patients who are well known to the clinician), the logistical challenges of using video due to high variability in appointments, problems accessing video call technology in a busy and complex work environment, and internet connectivity and local information technology helpdesk support difficulties [3].

Tweets about accessing remote consultations were more common than those referring to care quality. This could suggest that access is more pertinent to people or that there are more issues with accessing remote care compared to the quality of such care. However, this could simply reflect the limited number of characters for tweets, making it difficult to express views about care quality. Previous research that examined the content of tweets about hospitals showed that comments describing care were in the minority with various other topics being discussed [24].

Examining the sentiment of tweets about remote consultations over time revealed an interesting pattern in the data. The proportion of positive tweets regarding access to, and quality of, remote care was higher in the immediate period following the COVID-19 outbreak (March-May 2020) when compared to the time before the COVID-19 onset, and the time when restrictions from the first lockdown eased (June-October 2020). This is perhaps surprising as it might be expected that people would be less positive immediately following the lockdown when some services were in a state of flux and people would be unfamiliar with navigating new approaches to care delivery. Analysis of the tweets at different time periods provides insufficient detail to draw strong conclusions on the reason for this finding. One explanation might be that people were more understanding of the changes to care delivery initially when services were perceived to be under pressure and/or when changes were regarded to be temporary. There appeared to be several tweets posted in the later period since the pandemic (June-October 2020) that highlighted people’s frustrations that face-to-face consultations were still not being widely conducted despite the easing of restrictions.

Another explanation could relate to the fall in the number of people that sought health care during the initial period following lockdown (March-May 2020). With less people seeking health care during this period, there may have been greater availability of remote primary care appointments, resulting in a more positive experience for those people that did seek health care. This suggestion appears to be supported, in part, by experimental statistics on the length of time between booking and appointment dates. Over 60% of consultations took place on the same day as requested in April and May 2020, whereas there was a monthly downward trend subsequently, falling to 41% in October 2020 [2]. Furthermore, some patients may have decided to wait until they could have a face-to-face consultation, but then resorted to having a remote appointment in the later period (June-October) when it became clear that the virus was still having an impact on care delivery. These patients may have been less positive about the care being delivered remotely if their preference had been for face-to-face interaction.

Tweets that were negative about accessing care remotely centered on the difficulties of booking an appointment, lengthy waiting times for an appointment, and a lack of specific appointment times for phone consultations. These were noted in tweets posted both before and since COVID-19, although the volume of such tweets increased after March 2020. Results from the GP Patient Survey (prior to COVID-19) have shown a downward trend since 2012 in the proportion of patients reporting that it was “easy” to get through to their GP practice on the phone [25]. During March 2020, GP practices were advised by NHS England to triage patients before an appointment was made and to provide care remotely as much as possible [22]. Although practices moved toward more appointments being delivered remotely, our research shows that the issues surrounding the booking of appointments remained unchanged from those associated with a face-to-face appointment. Difficulties in getting through to the GP practice on the phone and issues with online appointment booking systems were frequently cited. The approach of triaging patients was tweeted about with mixed views; while some supported the need to triage patients, others were frustrated that decisions about clinical need appeared to be taken by GP practice receptionists.

The benefits of accessing care remotely were highlighted in the data. The efficiency of getting a remote appointment, and the convenience and safety of not needing to go to the GP practice were noted in tweets, with some people calling for remote consultations to be maintained in the long-term. Other studies have shown similar findings with patients valuing the convenience and time saved by video consultations when compared to face-to-face consultations [5,26,27]. Reduced travel time/expenses and convenience were also highlighted as benefits of telephone consultations by patients experiencing hospital-based telemedicine [28].

The contrast in views and experiences of accessing remote consultations may in part reflect differences between practices in the approach to appointment management and how well set up they were to deliver remote care prior to the pandemic. The Care Quality Commission found that some providers, especially larger ones, were able to move to remote consultations more easily due to already having the right technology in place [29].

There was a mix of views on the quality of care provided in remote consultations, although a higher proportion of tweets had a positive sentiment. A common positive theme, particularly immediately after the onset of COVID-19 (March-May 2020), was the ease and efficiency in which prescriptions had been issued. There is some evidence of a rapid increase in the prescribing of new medications for remote GP appointments, and it has been suggested that this may be the result of GPs being more cautious and prescribing medication “just in case,” or due to a shift in the case mix where more patients with new symptoms accessed remote appointments compared to face-to-face consultations [21]. Other positive aspects about the quality of care delivered remotely were quick referrals to secondary care, receiving follow-up information, and the interpersonal skills of health care professionals. A study conducted by Oxleas NHS Foundation Trust between March and July 2020 showed that patients reported receiving the same level of care and treatment during their remote appointment as they had received previously in face-to-face appointments, although it was noted that there was a preference for being seen face-to-face [5]. Further research is required to determine people’s willingness to receive remote care instead of face-to-face appointments in the longer term.

Tweets that were less positive about the quality of remote care implied that the standard of care was not as high as in face-to-face consultations, with a lack of visuals and physical examination being highlighted. Concerns were expressed by a small number of Twitter users that their condition had not be diagnosed or treated correctly via a telephone appointment. These findings support research carried out before COVID-19, which showed that, compared to face-to-face appointments, patients were less positive about the care received via remote consultations [25,30,31]. For instance, the 2020 GP Patient Survey (fieldwork January-March 2020, before the pandemic) showed that compared to face-to-face appointments, patients who had received a telephone appointment were 2% less likely to have their needs met, 4% less likely to say they were given enough time, and 4% less likely to feel that any mental health needs were recognized or understood [21].

The mixed attitudes toward remote care evident from our Twitter data support the view that although care delivered remotely can offer efficiency and convenience for patients and allows easier access for some groups of people, face-to-face consultations are more appropriate for others, or for certain conditions or situations [1,5,32-34]. It is important that patients can choose the type of consultation that best suits their needs and that the increased use of technology for delivering care does not become a barrier for some.

### Policy Implications

There has been a rapid shift to delivering health care remotely since the COVID-19 pandemic, and it is important to learn from those who have been at the forefront of experiencing such changes. Being able to deliver care remotely may have potential for improving efficiencies in health and social care systems, but further research is needed. Our analysis of Twitter comments has shown mixed attitudes and experiences to these changes. The finding that overall sentiment about remote care was more positive in the early stages of the pandemic but has since declined is important. Although the reasons for this can only be speculated, it does emphasize the need for a continued examination and understanding of people’s experiences as remote services continue to evolve.

There have been calls by policymakers for the increased use of remote care delivery to continue after the pandemic [35], although some GPs have found the high levels of remote care delivery a strain, have missed face-to-face contact with patients, and have been concerned about the clinical risk associated with delivering care in this way [23]. It has also been argued that sufficient funding and technical infrastructure are required to ensure that the increase in remote care provision can be successfully embedded [1,26]. A report by the Care Quality Commission noted that information technology systems were sometimes a barrier for patients and providers, with a lack of equipment in some sectors and some patients finding it difficult to adapt to using the new digital systems [29]. Although our research has highlighted some Twitter users with positive views about remote care and the benefits that it can offer, this study also shows where improvements are needed. Further research is needed to explore the challenges and barriers associated with remote care delivery to inform the future planning and delivery of remote care. Despite the shift to more consultations now being delivered remotely, the difficulties surrounding booking and getting an appointment remain an issue for some. Health care providers should offer specific appointment times for telephone consultations, not only to improve patient’s experiences but also to minimize appointments being missed and to protect patient confidentiality.

Our research also found that some people were negatively anticipating care being delivered remotely, including confusion as to why appointments requiring tests or physical procedures had been booked as a telephone consultation. Some of this confusion likely reflects initial difficulties experienced by providers due to the speed at which remote care was implemented. However, it does suggest that providing people with more information about how remote consultation works may improve public attitudes and acceptance, in addition to giving patients a better experience and avoiding the anxieties leading up to a remote care appointment.

### Limitations

There were some limitations of using Twitter comments to understand views on care being delivered remotely. The data are limited to internet users who engage with this social media platform and who tweeted between January 2018 and October 2020, and therefore do not represent the views of all health care users. Although the number of internet nonusers (ie, adults who have either never used the internet or have not used it in the last 3 months) has been declining over time, 10% of the adult UK population in 2018 were internet nonusers [36]. There is a “digital divide” as internet use and digital skills vary for different groups of the population; for example, internet nonusers are disproportionately disabled, women, those aged over 75 years, and those who are not in employment [36,37]. There are therefore concerns that the delivery of care remotely may negatively impact some groups of people more than others, such as those with limited digital literacy and/or lack of access to technology [38,39]. This is difficult to examine in an analysis of Twitter comments, as such groups are less likely to use social media platforms such as Twitter to share their views.

Since tweets are unprompted, despite carefully planned search terms, the extracted data are not always relevant. Over 1000 tweets were extracted using three search terms, but more than one third of these comments were irrelevant to the research question. Several tweets were also coded into themes that did not directly relate to understanding people’s views and attitudes of remote care delivery. The relatively low number of relevant comments is important to consider when comparing the proportion of tweets with a negative/neutral/positive sentiment within themes. Although there was a sufficient volume for identifying overall themes and trends, there was an insufficient level of detail to elucidate the reasons for some findings, highlighting the need for further research.

Another limitation of the study was the inability to examine any variations in views of remote care delivery by geographical region or by demographic factors such as age or gender. This was either due to the relatively low volume of relevant tweets extracted from our search terms (for geographic comparisons) or because such information was not available in the metadata (gender or age). Similarly, although one of the search terms sought to explore any changes in care delivery for people living with long-term conditions, there was an insufficient number of tweets referring to particular long-term conditions to allow for analysis by health condition. Long-term conditions are more prevalent in older and in more deprived groups [40], which are the same groups experiencing greater digital exclusion. As mentioned previously, the “economically inactive” are the most likely to be internet nonusers, particularly adults on long-term sick leave (due to health conditions that last 12 months or more) or who are disabled [36]. It is therefore likely that those with long-term conditions were underrepresented in the tweets analyzed in this study.

As previously mentioned, most of the tweets extracted were about GP consultations, and therefore our research does not provide as much insight into Twitter users’ views of remote secondary care. There were also very few tweets that referenced alternative methods of remote care delivery other than telephone or video consultations. This may reflect that other approaches such as online messaging systems or live chat are not yet being widely used to deliver remote care. It may, however, point toward a limitation of the search terms used, which did not include words such as “live chat” or “message/messaging,” although the word “online” was used.

### Conclusion

Using Twitter data to address our research questions was beneficial for providing rapid access to Twitter users’ attitudes about remote care delivery at a time when it was difficult to conduct primary research due to the COVID-19 pandemic. This approach allowed us to examine Twitter users’ views and experiences of remote care, and to explore shifting attitudes at a time of rapid changes in care delivery. However, we recognize that our findings do not represent the views of all health care users, and further research using alternative methodologies such as in-depth interviews with patients could complement our findings to provide further insight into people’s experiences of receiving remote care.

## Abbreviations

API: application programming interface
GP: general practitioner
NHS: National Health Service
NIHR: National Institute for Health Research
